# Relationship between learning styles and clinical competency in nursing students

**DOI:** 10.1186/s12909-024-05432-z

**Published:** 2024-04-26

**Authors:** Seyed Kazem Mousavi, Ali Javadzadeh, Hanieh Hasankhani, Zahra Alijani Parizad

**Affiliations:** 1 Department of Nursing, Abhar School of Nursing, Zanjan University of Medical Sciences, Zanjan, Iran; 2 Ph.D Candidate in Nursing, School of Nursing and Midwifery, Guilan University of Medical Sciences, Rasht, Iran; 3 Department of Nursing, School of Nursing and Midwifery, Guilan University of Medical Sciences, Rasht, Iran

**Keywords:** Learning, Clinical competence, Students, Nursing

## Abstract

**Background:**

The acquisition of clinical competence is considered the ultimate goal of nursing education programs. This study explored the relationship between learning styles and clinical competency in undergraduate nursing students.

**Methods:**

A descriptive-correlational study was conducted in 2023 with 276 nursing students from the second to sixth semesters at Abhar School of Nursing, Zanjan University of Medical Sciences, Iran. Data were collected using demographic questionnaires, Kolb’s learning styles, and Meretoja’s clinical competence assessments completed online by participants. Data were analyzed using SPSS version 16, employing descriptive statistics and inferential tests (independent T-test, ANOVA, Pearson correlation) at a significance level 0.05.

**Results:**

The predominant learning styles among nursing students were divergent (31.2%), and the least common was convergent (18.4%). The overall clinical competency score was 77.25 ± 12.65. Also, there was a significant relationship between learning styles and clinical competency, so the clinical competency of students with accommodative and converging learning styles was higher. (*P* < 0.05).

**Conclusion:**

The results of this study showed the association between learning styles and clinical competence in nursing students. It is recommended that educational programs identify talented students and provide workshops tailored to strengthen various learning styles associated with enhanced clinical competence.

## Introduction

Clinical competency is a multifaceted and nuanced concept that has been extensively explored and examined from various perspectives in recent years [1]. Its significance is underscored by the World Health Organization (WHO), which has identified the assessment and enhancement of nurses’ competencies as fundamental principles to uphold the quality of care. WHO defines nurses as competent when they can fulfill their professional responsibilities at the appropriate level, grade, and standard [2]. Factors such as evolving healthcare systems, the imperative for safe and cost-effective services, heightened community awareness of health issues, escalating expectations for quality care, and the demand for skilled healthcare professionals have elevated the importance of clinical competence in nursing and related fields [3]. Clinical competency is considered the ultimate objective and benchmark of nursing education effectiveness [4]. Notably, clinical education constitutes a pivotal component of nursing training, with over half of nursing programs dedicated to practical training [5]. A nursing student’s ability to become a proficient nurse at the bedside hinges on acquiring essential skills during their academic journey and attaining requisite qualifications [6]. Scholars argue that continual efforts to enhance educational quality are essential for upholding nursing care standards and improving clinical competence [7, 8]. Understanding how learners acquire knowledge is crucial for enhancing educational quality, with learning styles playing a pivotal role in this process [9].

Learning refers to the relatively enduring behavioral changes from experiences [10]. Learning styles, a concept widely embraced by educational theorists in recent decades, pertain to individuals’ distinct approaches to processing information and acquiring knowledge [11]. These styles encompass cognitive and psychosocial traits that are relatively stable indicators of learners’ engagement with and response to their learning environments [12]. Among the myriad theories on learning styles, Kolb’s learning theory is particularly influential [13]. According to Kolb, learning can be categorized into four primary modes: concrete experience, abstract conceptualization, reflective observation, and active experimentation, yielding four learning styles—converging, diverging, assimilating, and accommodating [14].

People with a converging learning style excel at problem-solving, decision-making, and practical application by engaging in abstract conceptualization and active experimentation [15]. Diverging learners thrive on experiencing and closely observing situations, possessing a unique ability to view scenarios from multiple perspectives and synthesize information into a cohesive whole [16]. The assimilating learning style is characterized by a preference for deep thinking and thorough examination, with individual’s adept at organizing information and employing abstract concepts to comprehend complex situations [13]. Accommodating learners learn best through hands-on experiences and activities, demonstrating proficiency in working with tangible objects and gaining new insights through practical engagement [10]. Learning styles are essential in nursing education because the primary mission of nursing education programs is to train nurses who have the necessary knowledge, attitude, and skills to maintain and improve the health of society members, and in other words, have sufficient competence in providing their job duties [17].

So far, separate studies have been conducted on nursing students’ learning styles and clinical competency [4, 8, 13, 17]. However, the relationship between these two concepts has received less attention from researchers. The first step in ensuring students’ academic success is to determine their learning style [11]. Professors’ awareness of the student’s learning styles and the relationship between these styles and the level of clinical competency provide a favorable opportunity to identify the styles with higher clinical competency and encourage students to use them as much as possible. Considering this importance, the researchers decided to design and implement the present study to investigate the relationship between learning styles and clinical competency in nursing students.

## Materials and methods

### Study design and sampling

This study was a descriptive-correlational study conducted in 2023, investigating the relationship between learning styles and the clinical competency of undergraduate nursing students. The research involved all second to sixth-semester undergraduate nursing students from the Abhar School of Nursing affiliated with Zanjan University of Medical Sciences, Iran. Sampling was carried out as a census, with 276 students selected to participate in the study. The inclusion criteria included willingness to participate in the study, full-time employment in nursing, no prior clinical work experience, and no reported psychiatric diseases or medication use. Incomplete questionnaire completion was set as an exclusion criterion.

### Instruments

The data collection tools included demographic questionnaires, Kolb’s learning styles questionnaire, and a modified Meretoja nursing clinical competency questionnaire. The demographic questionnaire gathered age, gender, marital status, semester, Grade Point Average (GPA), and interest in nursing.

The data collection tools included demographic questionnaires, Kolb’s learning styles questionnaire, and a modified Meretoja nursing clinical competency questionnaire. The demographic questionnaire gathered information such as age, gender, marital status, semester, Grade Point Average (GPA), and interest in nursing.

Kolb’s III learning styles questionnaire comprised 12 questions with four options each, requiring the student to select the option most similar to them. Each option represented one of the four main learning methods: concrete experience (CE), reflective observation (RO), abstract conceptualization (AC), and active experimentation (AE). Scores for the four learning styles were obtained from the total questions across the sections. By subtracting scores, two dimensions (AC - CE) and (AE - RO) were derived, determining the student’s learning styles as converging, diverging, accommodating, or assimilating [18]. This questionnaire has been used in various studies over the past 30 years, demonstrating validity and reliability. In Iran, Ghahrani et al. used the internal consistency method to determine the reliability of the questionnaire. They obtained Cronbach’s alpha of 71% in concrete experience, 68% in reflective observation, 71% in abstract conceptualization, and 71% in active experimentation [19]. Also, In the present study, the reliability value of this questionnaire was determined using Cronbach’s alpha method of 0.94.

Meretoja’s revised nursing clinical competency questionnaire contained 47 items across 5 areas of clinical competency: assisting patients (7 skills), teaching and guidance (12 skills), diagnostic measures (8 skills), therapeutic measures (5 skills), and occupational responsibilities (15 skills). Skills were rated on a four-point Likert scale, assessing the degree of skill utilization [20]. This questionnaire was psychometrically evaluated in Iran by Bahreini et al., and its validity was qualitatively determined at the optimal level, and its reliability was determined between 70 and 85%. Also, in this study, the reliability value of this questionnaire was determined using Cronbach’s alpha method of 0.91 [21].

### Data collection and statistical analysis

Following ethical approval and research permission, questionnaires, consent forms, and contact information for the researchers were provided to students online through the Porsline system (www.porsline.ir) for completion. Data analysis was performed using SPSS version 16 software, employing descriptive (frequency, percentage, mean, and standard deviation) and inferential (independent T-test, ANOVA, Pearson correlation) statistics at a significance level of 0.05.

## Results

Out of 276 participants, 10 students were excluded due to incomplete questionnaire responses, leaving 266 participants for analysis. The average age of students was 22.33, with 63.4% being female. Most participants were single (79.2%), and 46.2% had a GPA between 16 and 18. Also, 80.7% of students declared that they are interested in nursing. Then, the results of the questionnaires on learning styles and clinical competency were examined. Based on this, the findings showed that divergent (31.2%) and convergent (18.4%) styles were the study participants’ most and least-used learning styles, respectively. Also, the overall students’ clinical competence score was 77.25 ± 12.65 (Table 1).

**Table 1:** The frequency of learning styles and the level of clinical competency of the participants

Variables			
**Learning styles**	**N (%)**	**Clinical competency**	**Mean (SD)**
Diverging	83 (31.2)	Assisting patients	12.16 (1.24)
Accommodating	75 (28.2)	Teaching and guidance	19.26 (1.73)
Assimilating	59 (22.2)	Diagnostic measures	13.63 (1.17)
Converging	49 (18.4)	Terapeutic measures	7.23 (1.02)
		Occupational responsibilities	24.97 (2.13)
		Total score	77.25 (12.65

The relationship between the participants’ learning styles and clinical competency was examined in the next step. Initially, the study data underwent a normality assessment. The Kolmogorov-Smirnov test results indicated that parametric statistical tests were applicable (*p* > 0.05). Subsequently, an ANOVA test was conducted to explore the relationship between learning styles and clinical competency, revealing a significant association between learning style and clinical competency with moderate effect size (*p* < 0.05) (Table 2).

**Table 2:** Relationship between learning styles and clinical competency in students

Variables	Learning Styles				Test	P- value	Effect size (Cohen’s F)
	Diverging	Accommodating	Assimilating	Converging			
**Clinical competency** Mean (SD)	79.46 (10.42)	93.72 (11.12)	52.23 (9.12)	84.67 (12.12)	**ANOVA**	**<0.001**	**0.21**

The correlation between demographic variables, learning styles, and student clinical competency was investigated in the final phase of analyzing the findings. Parametric independent t-tests, Pearson’s correlation coefficient, and ANOVA were employed for this analysis. The results indicated that none of the learning styles exhibited a statistically significant relationship with the demographic characteristics of the participants (*p* > 0.05). However, a significant correlation was observed between participants’ demographic variables, such as age, academic semester, GPA, and interest in nursing, and their clinical competencies (*p* < 0.05) (Table 3).

**Table 3:** Relationship between demographic variables with learning styles and clinical competency of the participants

Demographic characteristics	Clinical competency	Diverging	Accommodating	Assimilating	Converging
Age	0.016* (r = + 0.31**)**	0.761** (F: 0.039)	0.549** (F: 0.082)	0.145** (F: 1.18)	0.340** (F: 0.095)
Gender	0.124*** (t: 0.232)	0.312** (F: 0.109)	0.097** (F: 2.02)	0.184** (0.098)	0.077** (F: 2.55)
Marital status	0.644*** (t: 0.243)	0.146** (F: 1.17)	0.417** (F: 0.127)	0.138** (F: 1.42)	0.603** (F: 0.067)
Semester	< 0.001** (F: 12.40)	0.260** (F: 0.176)	0.109** (F: 1.12)	0.078** (F: 2.85)	0.133** (F: 1.02)
GPA	< 0.001** (r = + 0.67**)**	0/091** (F: 1.66)	0.257** (F: 0.178)	0.545** (F: 0.086)	0.104** (F: 1.17)
Interest to nursing	< 0.001*** **(t: 3.93)**	0.321** (F: 0.115)	0.790** (F: 0.033)	0.711** (F: 0.040)	0.222** (F: 0.240)

## Discussion

This study explored the relationship between learning styles and clinical competency in undergraduate nursing students. The research initially focused on examining the variables and subsequently explored their interrelation. According to this, the most prevalent learning style among nursing students was divergent. This finding aligns with the outcomes of various domestic studies like Mehni et al. [22] and Shirazi et al. [16], as well as numerous international studies such as those by Campos et al. in Brazil and the United States [23], Nosheen in Pakistan [24], Madu et al. in Nigeria [25], and AbuAssi et al. in Saudi Arabia [26]. It should be said that the dominant abilities of people with divergent styles are concrete experience and reflective observation. They see the situation from multiple angles, emphasize brainstorming and generating ideas, have a strong imagination, are more sensitive to values, respect the feelings of others, and listen with an open mind and without bias [5]. Therefore, these people have high cultural interests and are more inclined towards humanities fields such as sociology, psychology, counseling, and nursing.

Upon reviewing studies in this field, it is concluded that findings often vary. They can be influenced by factors including individual student traits, educators’ teaching styles, learning environments, and tasks [12]. Also, this study noted no significant association between learning styles and participants’ demographic characteristics, consistent with similar research in the field [22, 23]. In this regard, Dantas et al. emphasized that learning styles predominantly reflect individuals’ traits and are minimally impacted by demographic variables [12].

Furthermore, the clinical competency level of nursing students was reported to be at an average level, consistent with findings from studies conducted in Iran [6, 27, 28] and other countries [29–31]. Some studies, however, have yielded differing results compared to the present study. For instance, Ghafari et al. [1], Katebi et al. [32], and Fung et al. [33] found that nursing students participating in their studies exhibited a higher level of clinical competency. Notably, participants in all three mentioned studies were in their final year of study undergoing the arena course. Hence, the emphasis on passing diverse training units and gaining more clinical experiences could justify the high clinical competency score achieved. Also, In the present study, the relationship between academic semesters and students’ clinical competency was significant, which confirms the above argument. Conversely, certain studies have reported a lower level of clinical competency among nursing students. For example, Getie et al. found that only one-third of nursing students demonstrated acceptable clinical competency [34]. These discrepancies in findings can stem from the questionnaire and the data collection method used. Notably, Getie et al. evaluated students’ clinical competency through assessments by cooperating nurses rather than self-assessment. Additionally, adjusting the questionnaire averages to reflect higher clinical competence could impact the reported competency levels. Various factors, such as individual, environmental, organizational, and educational characteristics, influence the acquisition of clinical competency in nursing students [2]. Hence, diverse study outcomes exist in this field. Also, besides academic semesters, relationships were observed between age, GPA, interest in the field, and nursing students’ clinical competency. These results align with the findings of many studies in this area [27–29, 32, 33]. Older students are often in their final semesters, potentially showcasing higher clinical competency due to their exposure to clinical environments. Madjid et al. highlighted in their study that good grades obtained by learners in any field indicate their interest in the subject. This aspect holds particular significance in nursing—a complex and demanding profession where success hinges on a genuine interest and academic excellence [35].

Also, the study revealed a significant relationship between nursing students’ learning styles and clinical competency. According to this, students employing accommodating and converging learning styles reported heightened levels of clinical competency. In their study, Ebrahimi Fakhar et al. noted that medical students utilizing reflective observation and active experimentation learning methods exhibited enhanced clinical competency upon course completion. As these attributes align with accommodative and convergent learning style characteristics, the present study’s results are consistent with these findings [36]. Moon et al. found that nursing students with converging and accommodating styles reported increased competence in clinical practices [37]. Similarly, Lundell Rudberg et al. revealed that nursing students with these learning styles demonstrated greater professional responsibility, a key aspect of clinical competence. This correlation indirectly supports the present study’s outcomes [38]. Generally, it can be said that people with convergent and accommodating learning styles typically gravitate toward practical learning. They are inclined towards hands-on activities, deriving their learning mainly through experience and active participation [12, 15]. Therefore, they are expected to engage more in clinical settings, fostering heightened clinical competency. Furthermore, the study’s latest findings indicated that students with an assimilating learning style exhibited lower levels of clinical competency, aligning with Moon et al.‘s study [37]. Similarly, Figueiredo et al. explored nurses’ learning styles based on Kolb’s theory in qualitative research, noting that nurses with assimilating learning styles are inclined towards abstract and subjective concepts over practical content and may have less enthusiasm for immersive clinical environments [39].

### Limitations

One limitation of this study was the potential for inaccuracies in questionnaire completion due to the electronic data collection method and the extensive number of questions. To address this concern, participants were provided with researchers’ contact details for clarifications during data collection. Another limitation was the reliance on self-report tools and the omission of considering individuals’ personality traits in measuring the research variables, factors beyond researchers’ direct control.

## Conclusion

The study’s findings underscore the relationship between learning styles and clinical competency in undergraduate nursing students. Therefore, considering the high importance of acquiring clinical competency in these students, it is recommended that educational administrators identify students prone to declining clinical competency based on their learning styles and organize workshops to enhance styles associated with superior clinical competency. Also, Given the complexity of learning styles and clinical competency as constructs, future studies may benefit from exploring additional theories and tools to delve deeper into these concepts, employing qualitative or mixed-method approaches for comprehensive analysis.

## Data Availability

Data is provided within the manuscript or supplementary information files.
